# Alternative Approach to the Synthesis of Vinyl Ester Resins—Composites and Their Biomedical Application

**DOI:** 10.3390/polym17243251

**Published:** 2025-12-06

**Authors:** Przemysław Pączkowski, Karolina Głogowska, Małgorzata Miazga-Karska, Grażyna Ginalska, Barbara Gawdzik

**Affiliations:** 1 Department of Polymer Chemistry, Institute of Chemical Sciences, Faculty of Chemistry, Maria Curie-Skłodowska University, Gliniana 33, 20-614 Lublin, Poland; barbara.gawdzik@umcs.pl; 2 Department of Technology and Polymer Processing, Faculty of Mechanical Engineering, Lublin University of Technology, Nadbystrzycka 36, 20-618 Lublin, Poland; k.glogowska@pollub.pl; 3 Chair and Department of Biochemistry and Biotechnology, Faculty of Pharmacy, Medical University of Lublin, Chodzki 1, 20-093 Lublin, Poland; malgorzata.miazga-karska@umlub.pl; 4 Faculty of Health Sciences, Vincent Pol University in Lublin, Choiny 2, 20-816 Lublin, Poland; g.ginal@poczta.onet.pl

**Keywords:** vinyl ester resin, wood flour, composite, cytotoxicity, antimicrobial

## Abstract

The paper presents the results of research on composites containing wood flour and vinyl ester resins as matrices. One of the resins was a commercially available vinyl ester resin (VER) based on bisphenol A, while the other (VPE)—not containing bisphenol A, was obtained by us in an innovative way protected by a patent, whereas light yellow wood flour (WF) powder was obtained from spruce (*Picea*) and fir (*Abies*). Due to the fact that vinyl ester resins are characterized by large mechanical and chemical resistance, they are used mainly in the form of composites for the production of everyday products. To verify the possibilities of their biomedical applications, our studies focused on the evaluation and comparison of cytotoxicity of both resins using human skin fibroblasts and their resistance to bacterial bio-film adhesion for the aerobic Gram-positive bacteria *Staphylococcus aureus* ATCC 25923, *Enterococcus faecalis* PCM 896 (Polish Collection of Microorganisms), and the aerobic Gram-negative bacteria *Escherichia coli* ATCC 25992.

## 1. Introduction

Products made of epoxy, polyester, and vinyl ester resins are commonly used in households, public buildings and various industries. In order to reduce the cost of production without significant reduction in utility properties of the products, various types of fillers are used. When obtaining composites, biofillers have often been taken into account because they can serve as centers of biodegradation. Such composite materials are commonly used in the production of yachts, sailboats, bathtubs, shower cabins, medical equipment, houses, and other similar applications, as well as in elements made of wood, such as parquet or wainscoting.

Pączkowski et al. presented the research on the antibacterial resistance of unsaturated polyester resin (UPR) composites [1]. As follows from the results, microorganisms survive on inanimate “touch” surfaces for a long time, and only the use of colloidal silver prevents bacterial colonization.

This can be particularly problematic in healthcare, where the patient’s immunity is more vulnerable to infections. Touch surfaces in hospital rooms can serve as a source or a reservoir for bacterial expansion. The most common pathogens found in healthcare settings are aerobic Gram-negative *Escherichia coli* (*E. coli*) and Gram-positive *Staphylococcus aureus* (*S. aureus*), as well as *Enterococcus faecalis* (*E. faecalis*) bacteria. These bacteria are responsible for severe nosocomial infections [2]. Preparation of polymeric materials with potential antimicrobial properties may be the answer to the problem [3,4,5,6].

Currently, the antibacterial properties of composites based on vinyl ester resins are being investigated. To prepare the materials, a commercially available vinyl ester resin and an analog synthesized in this paper, without using a bisphenol-A derivative as a precursor, were used.

Vinyl ester resins (VERs) are a specific type of polyester resin. Similar to unsaturated polyester resins, they are available as solutions in a crosslinking monomer, usually styrene. In this case, instead of unsaturated polyester, the addition product of epoxy resins with meth/acrylic acid is dissolved in the crosslinking monomer. Compared with UPR, they cure rapidly [7]. They are usually applied when improved strength and chemical resistance are required in some applications. As mentioned above, vinyl ester resins offer toughness and chemical resistance properties that are generally superior to unsaturated polyesters [8]. The epoxy resin backbone used in making vinyl ester resins confers toughness and greater tensile elongation properties to these resins. The chemical resistance of these resins is due to the presence of phenyl ether bonds instead of ester groups. The latter moieties are much more resistant than ester linkages to degradation in many chemical environments, and especially in high pH alkaline situations. The ester linkages in a vinyl ester resin are present only at the end of the molecule, which minimizes the number of ester linkages that can be chemically attacked. Furthermore, if the vinyl ester resin molecule is terminated with methacrylate groups, the spatially large methyl group pendant on the methacrylate group sterically shields the ester linkage from chemical attack [9].

Vinyl ester resins are largely applied as matrix systems in high-performance fiber-reinforced composites. Technologically, vinyl ester resins are a kind of compromise between unsaturated polyester resins and epoxy resins [7]. The disadvantage of this type of resin is the fact that its chains contain derivatives of bisphenol-A, which is commonly considered to be toxic [10].

To eliminate this disadvantage of vinyl ester resins, an analogous VPE resin based on unsaturated polyesters was synthesized, in which carboxyl end groups were reacted with glycidyl methacrylate and then dissolved in styrene. The new resin contains vinyl end groups similar to those of commercial VERs. However, its chemical structure does not include fragments derived from toxic bisphenol A and consists solely of chains typical of unsaturated polyesters.

Unsaturated polyesters are synthesized from glycols and a mixture of saturated and unsaturated carboxylic acids, primarily phthalic acid (or its anhydride) and maleic acid. The main chain fragment of the resin was formed by a polycondensation reaction between the acidic components (phthalic and maleic anhydride) with bis(2-hydroxyethyl)terephthalate (BHET), which served as the hydroxyl-containing component. Complete VPE resin chains were obtained after the addition of glycidyl methacrylate to the terminal (end) carboxyl groups of the polyester. It is noteworthy that bis(2-hydroxyethyl)terephthalate was derived from the glycolysis of waste poly(ethylene terephthalate) (PET) obtained from water bottles.

Wood flour (WF) was used to obtain composites based on vinyl ester resins. Wood waste is a frequently used filler in composites [1,11]. Numerous works claim that some types of wood have antibacterial properties [12,13,14,15]. It is assumed that wood flour acts as an “antibacterial promoter” for polymeric materials [16].

In the presented work, composites based on two different vinyl ester resin matrices modified with the same content of the wood flour from spruce (*Picea*) and fir (*Abies*) trees were used for comparative studies. Moreover, the influence of the presence of filler on the antimicrobial properties, as well as the mechanical and surface properties of the obtained materials, was investigated.

## 2. Experimental

### 2.1. Materials

The bisphenol A-based vinyl ester resin (VER), Estromal VE-06 (Brookfield viscosity at 25 °C: 752 mPas; non-volatile content: 74%), produced by Lerg S.A. (Pustków, Poland), was used for the preparation of one group of composites with wood flour.

To prepare the second group of composites, a resin synthesized in this study, an analog of unsaturated vinyl ester resin, was used. Its synthesis involved two main steps. First, an unsaturated polyester was produced by polycondensation of phthalic and maleic anhydrides with bis(2-hydroxyethyl)terephthalate (BHET). In the second step, the terminal-carboxyl groups of the polyester (acid number: 53.4 mg KOH g^−1^) underwent an addition reaction with glycidyl methacrylate (GMA), introducing vinyl groups at the ends of the polyester chains.

Phthalic and maleic anhydrides were bought in P.P.H. Standard Sp. z o.o. (Lublin, Poland). Bis(2-hydroxyethyl)terephthalate (BHET), as a product of poly(ethylene terephthalate) (PET) glycolysis, was supplied by Lerg S.A. (Pustków, Poland). Butylstannoic acid (Fascat 4100) used as an esterification catalyst was purchased from PMC Organometallix (Carrollton, TX, USA).

Methyl ethyl ketone peroxide (MEKP, Luperox DHD-9) as an initiator, glycidyl methacrylate (GMA) as a modifying agent, ethyltriphenylphosphonium bromide (ETPB) as a catalyst, tert-butylhydroquinone (TBHQ), and 1.4-naphthoquinone (NQ) as an inhibiting system were purchased from Sigma-Aldrich (St. Louis, MO, US).

The co-accelerator: *N*,*N*′-diethylaniline (DEA) was supplied by Fluka Chemie AG (Buchs, Switzerland). The 4% solution of an environmentally friendly polymeric cobalt accelerator was synthesized at the Department of Polymer Chemistry of Maria Curie-Skłodowska University (Lublin, Poland) [17].

Wood flour (WF) from JELU-WERK Ludwigsmühle Josef Ehrler GmbH & Co. KG (Rosenberg, Germany) was used as a filler for vinyl ester resins to obtain wood resin composites. JELUXYL light yellow powder originated from spruce (*Picea*) and fir (*Abies*). Softwood, which is an organic wood and plant fiber, consists mainly of compounds from cellulose and lignin (~90%) and moisture (~10%). The filler had pH = 5.5 and an Alpine air sieve fraction of 75 μm (~35%), 100 μm (~20%), and 180 μm (traces), respectively.

### 2.2. Synthesis of VPE

To a flask equipped with a mechanical stirrer and a thermometer 37.95% (296.24 g) phthalic anhydride, 25.12% (196.12 g) maleic anhydride, and 37.0% (289.16 g) bis(2-hydroxyethyl)terephthalate (BHET) were added, using a 37:63 *w*/*w* ratio of hydroxyl to acidic reagents, along with 0.02% (0.15 g) butylstannoic acid (Fascat 4100) as an esterification catalyst and 50 mL of xylene as an azeotropic agent. The reactants were stirred and heated to 170 °C, removing water in the process. After approximately 2.5 h, the polyester was cooled to 50–60 °C.

Then, 100 g of the obtained unsaturated polyester, with an acid number of 53.4 mg KOH, was placed in a flask equipped with a mechanical stirrer and a thermometer. After heating to 60 °C, a solution consisting of 33.9 g of glycidyl methacrylate (GMA), 0.339 g of inhibitor (1% relative to GMA; *tert*-butylhydroquinone + 1.4 naphthoquinone), and 5.09 mg of ETPB catalyst (ethyltriphenylphosphonium bromide) was added [18]. All the ingredients were mixed until a homogeneous mass was obtained at a stirrer speed of 800 rpm, after which the temperature was raised to 80 °C and maintained for 1 h.

The reaction progress was monitored by FT-IR spectroscopy, tracking the disappearance of epoxy bands. When a significant decrease in the intensity of the bands originating from the epoxy ring of GMA was observed, a second portion of catalyst (5.09 mg) was added, and the mixture was heated until the epoxy bands disappeared completely. After cooling to room temperature, the resulting polyester, containing vinyl groups at the chain ends (VPE), was dissolved in styrene as a crosslinking monomer at a mass ratio of 60% VPE to 40% styrene.

### 2.3. Resin Curing and Sample Preparation

To cure both VER and VPE vinyl esters, a system consisting of 1.0 wt.% of methyl ethyl ketone peroxide (MEKP, Luperox), 0.12 wt.% of a 4 percent polymer solution of cobalt, and 0.06 wt.% of *N*,*N*’-diethylaniline (DEA) was used. The same amounts of initiator and accelerator were applied for the preparation of WF/resin composites with a different filler amount.

To ensure good homogeneity, the prepared mixtures were well-mixed and degassed under vacuum. Then mixtures were poured into the cuboid-shaped molds composed of two glass panes coated with a thin anti-adhesive layer and separated by a 4 mm thick Teflon spacer.

All materials were cured under identical conditions: first at room temperature for 24 h, then at 80 °C for 1 h, followed by 3 h at 110–115 °C to complete the reaction. After curing, the cuboid-shaped samples were cut to the required dimensions for testing using a CNC milling machine MFG 8037P from Ergwind (Gdańsk, Poland).

## 3. Methods

### 3.1. Chemical Characterization

#### 3.1.1. IR Spectroscopy

The characteristic bands of the functional groups in the samples were determined using ATR/FT-IR spectroscopy. The spectra were recorded on a Bruker TENSOR 27 spectrometer (Ettlingen, Germany) over the frequency range of 600–4000 cm^−1^ with a resolution of 4 cm^−1^ at 32 scans per sample. Prior to the measurements, background spectra were also recorded for reference.

#### 3.1.2. NMR Spectroscopy

The ^1^H NMR and ^13^C NMR spectra were recorded using an NMR Bruker Avance 300 MHz spectrometer (Bremen, Germany). The chemical shifts were referenced to deuterated chloroform as an internal standard, with δ = 7.26 for ^1^H NMR, and δ = 77.28 for ^13^C NMR.

### 3.2. Mechanical Properties

#### 3.2.1. Hardness

The effect of the wood flour on the hardness of the wood-resin composites was evaluated using a Shore durometer 7206/H04 from Zwick (Ulm, Germany) according to the EN ISO 868:2003, at a standard temperature of 23 °C ± 2 °C [19]. The final hardness value was the arithmetic mean of five individual measurements (each taken after 15 s).

#### 3.2.2. Three-Point Bending—Flexural Properties

The mechanical properties of the vinyl esters and their wood composites, with the dimensions of 80 mm × 10 mm × 4 mm, were determined using a Zwick/Roell Z010 universal testing machine (Zwick GmbH Co, Ulm, Germany). A three-point bending flexural test was carried out at room temperature, with a support span of 64 mm, a speed of 5 mm min^−1^, and a load capacity of 10 kN. The flexural modulus ($E_{f}$), flexural strength ($\sigma_{f}$), and strain at break ($\varepsilon_{f}$) were determined. Measurements were performed in accordance with the EN ISO 178:2019, and the arithmetic average of five individual measurements was considered as the final result [20].

#### 3.2.3. Charpy Impact Strength

The Charpy impact test was performed according to EN ISO 179-2 using a 639F-type impact hammer (Cometech Testing Machines, Taizhong, Taiwan) with a pendulum maximum energy of 5 J [21]. The resin specimens with the dimensions of 80 mm × 10 mm × 4 mm were used for the impact test.

#### 3.2.4. Dynamic Mechanical Analysis

Dynamic mechanical analysis (DMA) of the resin-based composites was performed using a DMA-Q800 (TA Instruments, New Castle, DE, USA) equipped with a dual cantilever clamp. Specimens with dimensions of 65 mm × 10 mm × 4 mm were submitted to a sinusoidal deformation with an amplitude of 0.01 mm at a constant frequency of 1.0 Hz over a temperature range of −150 to 200 °C. The heating rate was maintained at 5 °C min^−1^. Single DMA measurements were performed according to EN ISO 6721-1:2019 [22].

The viscoelastic properties of the cured materials were estimated from the temperature-dependent changes in storage modulus ($E^{\prime }$), loss modulus ($E^{\prime \prime }$), as well as loss or damping factor (tan δ) at a constant frequency. The glass transition temperature ($T_{g}$) was identified as the maximum of the tan δ curve. Additionally, the full width at half maximum (FWHM) of the damping factor was determined.

To estimate the crosslinking density ($v_{e}$) of the cured unsaturated polyester resin, the following relation for thermosetting polymers was applied (Equation (1)) [23,24]:

(1) $$\nu_{e}=\frac{E_{e}^{\prime }}{3RT}$$

where $v_{e}$ is the crosslinking density, mol m^−3^; $E_{e}^{\prime }$ is the storage modulus at $T_{g}$ + 50 °C, i.e., the equilibrium storage modulus in the rubber zone, Pa; R is the universal gas constant, 8.314 m^3^ Pa mol^−1^ K^−1^; and T is the absolute temperature at $T_{g}$ + 50 °C, chosen to ensure the plateau zone, K.

### 3.3. Surface Properties

The surface analyses of the wood-resin composites were based on gloss measurements, wetting tests, and contact angle determination.

The surface gloss of the obtained materials was measured using a Zehntner ZGM 1110 triple-angle gloss meter (Zehntner GmbH Testing Instruments, Sissach, Switzerland), as described earlier [25]. The tests were conducted in accordance with the ASTM D2457 standard, and the arithmetic mean of ten measurements was taken as the final result [26].

The contact angle was determined using an OCA 15EC video-based optical contact angle measurement system (DataPhysics Instruments GmbH, Filderstadt, Germany) equipped with a DSA100S droplet shape analyzer (KRÜSS GmbH, Hamburg, Germany). The measurement was recorded with a CCD camera 5 s after depositing a 2 μL drop of distilled water. All contact angle tests were performed under controlled conditions: temperature 21 ± 1 °C and relative air humidity 30 ± 1%.

### 3.4. Bacterial Biofilm

#### 3.4.1. Bacterial Strains

The tiles of tested material samples were evaluated for their anti-biofilm character against aerobic Gram-positive *Staphylococcus aureus* ATCC 25923 and *Enterococcus faecalis* PCM 896 (Polish Collection of Microorganisms), as well as the aerobic Gram-negative *Escherichia coli* ATCC 25992. In the microbiological assay, the Mueller–Hinton broth (MH-broth) was used for aerobic strains, while the Brain-Heart Infusion broth (BHI-broth) was used for microaerobic strains. After 24 h of bacterial growth at 37 °C on the solid medium, an inoculum was prepared in 2 mL of 0.9% NaCl, adjusted to 0.5 McFarland turbidity standard 1.5 × 10^8^ CFU mL^−1^ (CFU: colony forming unit).

#### 3.4.2. Materials Seeding with Bacteria for Biofilm Determination

The square tiles of tested samples (resins and their WF composites), approximately 10 mm in diameter and 4 mm in height, were sterilized by briefly immersing them alternately in 1000 µL of water, 1000 µL of 70% ethanol, and 1000 µL of 0,9% NaCl. The dry samples were then transferred to the bottoms of 24-well polystyrene plates (CytoOne, Ocala, FL, USA). Subsequently, 1000 µL of BHI broth was added to each well containing square samples, or to the empty wells that served as the positive control of bacterial growth. Finally, 10 µL of the bacterial inoculum (1.5 × 10^8^ CFU mL^−1^) was added.

For the mono-species biofilm assay, the following strains were used: the aerobic Gram-positive *S. aureus* ATCC 25923 or *E. faecalis* PCM 896, and the aerobic Gram-negative *E. coli* ATCC 25992. Sterility controls (wells containing only MH- or BHI-broth) were included in all experiments.

To allow biofilm formation, i.e., the adhesion of planktonic bacterial cells forming the colonies on the biomaterial, the plates were incubated twice. Namely, the plates with aerobic bacteria were incubated for 48 h at 37 °C. All tests were performed in triplicate.

#### 3.4.3. CLS Microscopy Biofilm Visualization

The aim of the test was to visualize the viability and potential adhesion of bacterial cells to the polymeric materials (VPE pure or modified, VER pure or modified) in comparison with their adhesion to the control surface of a 24-well plate. The viability of bacterial cells and their adhesion to the sample surfaces were assessed using double fluorescent staining for both live and dead bacteria with the Viability/Cytotoxicity Assay kit for Bacteria LIVE/DEAD Cells (Biotium, Hayward, CA, USA) [27].

Material samples containing bacterial suspensions were prepared for confocal microscopy after the double-timing incubation period. First, the medium was removed from the wells, and the samples were gently washed twice with 500 µL of 0.9% NaCl to retain only the bacterial biofilm adhered to the material and to remove loosely attached planktonic cells. The samples were then transferred to fresh wells and filled with 500 µL of 0.9% NaCl and the LIVE/DEAD dye.

The dye solution was prepared by mixing 1 µL of dimethyl sulfoxide (DMSO) with 1 µL of Ethidium Homodimer III (EthD-III) in 8 µL of 0.9% NaCl. 5 µL of such obtained solution were added to the wells containing the material samples (or to empty wells used as a positive control for bacterial growth), each filled with 500 µL of phosphate-buffered saline (PBS). The samples were incubated for 20 min at room temperature in the dark, and bacterial colonies adhered to the material surfaces were visualized using a confocal laser scanning microscope (CLSM) equipped with dedicated software.

#### 3.4.4. Quantitative Biofilm Determination

In addition to visualization using CLSM, a quantitative assessment of biofilm formation was also performed. To determine the extent of biofilm on the polymer surfaces, the procedures described by O’Toole [28] were followed. After 48 h of incubation (double incubation) of the plate samples with bacterial strains, the medium was removed, and the samples were washed twice with 500 µL of fresh medium to remove planktonic cells and leave only the bacteria adhered to the surface.

The remaining cells, constituting the formed biofilm attached to the materials, were stained with 1 mL of 0.1% crystal violet (CV) for 10 min at room temperature to allow the visualization of the biofilm. The samples were then transferred to the fresh wells and washed twice with 500 µL of sterile water to remove any CV unbound to the bacteria. To solubilize the bound dye, each biofilm-coated sample was placed in a separate tube containing 1000 µL of 20% acetic acid and incubated for 15 min at room temperature. The tubes were subsequently sonicated for 2 min to disperse the biofilm.

Next, 200 µL of the obtained CV-acetic acid solution was transferred to a new 96-well plate for optical density (OD) measurement at 590 nm. For each sample, OD determination (200 µL taken from 1000 µL solution) was performed five times, and the average value was calculated.

For each group of materials, a negative control (sterility control consisting of the sample immersed in MH-broth) and positive growth controls (MH-broth in the wells of polystyrene plates with *S. aureus* ATCC 25,923 or *E. faecalis* PCM 896, or *E. coli* ATCC 25992) were included. The OD value of the positive controls was treated as 100% biofilm formation.

The results obtained in the experiment were analyzed for statistically significant differences (*p* < 0.05, n = 3) relative to the positive control of biofilm formation using an unpaired t-test (GraphPad Prism 5, Version 5.03 GraphPad Software, Inc., San Diego, CA, USA).

### 3.5. Cytotoxicity

#### 3.5.1. Quantitative Evaluation of Cytotoxicity

Cytotoxicity assessment was performed in accordance with the EN ISO 10993-5 [29]. Normal human skin fibroblasts (BJ cell line), obtained from the American Type Culture Collection (ATCC-LGC Standards, Teddington, UK), were seeded into 96-well plates in 100 μL of Eagle’s Minimum Essential Medium (EMEM, ATCC-LGC Standards, Teddington, UK) supplemented with 2% fetal bovine serum (Pan-Biotech GmbH, Aidenbach, Germany) at a density of 2 × 10^4^ cells per well. The cells were cultured for 24 h at 37 °C until reaching near confluence.

The growth medium was then replaced with 24 h extracts of the tested samples, prepared in accordance with the EN ISO 10993-12 standard [30]. Complete growth medium was used as a negative cytotoxicity control. After 24- and 48 h of incubation at 37 °C, the viability of BJ cells was assessed using the colorimetric WST-8 test (Sigma-Aldrich Chemicals, Poznań, Poland), following the manufacturer’s protocol. The test was performed in triplicate, and the optical density of the resulting solution was measured using a Synergy H1 Hybrid microplate reader (Agilent Technologies, Winooski, VT, USA).

The cytotoxicity results were analyzed for statistically significant differences (*p* < 0.05) between the tested groups using one-way ANOVA followed by Tukey’s multiple comparison test (GraphPad Prism 8, Version 8.01).

#### 3.5.2. Live/Dead Staining

Cell viability was further confirmed by Live/Dead double fluorescent staining of BJ cells following their exposure to the material extracts. Fibroblasts were seeded into 96-well plates in 100 μL of complete growth medium at a density of 2 × 10^4^ cells per well and cultured at 37 °C for 24 h. The growth medium was then removed and replaced with 24 h extracts of the tested materials, followed by 48 h incubation under the same conditions.

Subsequently, the BJ cells were stained using the Live/Dead double staining kit (Sigma-Aldrich Chemicals, Poland) according to the manufacturer’s instructions. The fluorescence staining kit consists of calcein-AM, which emits green fluorescence in viable cells, and propidium iodide, which emits red fluorescence in dead cells.

Fibroblasts’ viability was analyzed and visualized using a confocal laser scanning microscope (CLSM, Olympus Fluoview equipped with FV1000, Olympus, Tokyo, Japan). Multiple series of optical cross-sections (Z-stacks) were acquired using a UPLSAPO 10× objective lens. Optical sections were recorded at a resolution of 1600 × 1600 pixels. The final visualizations were reconstructed using ImageJ 2.15.0 (LOCI, University of Wisconsin, Madison, Wisconsin, USA).

## 4. Results and Discussion

### 4.1. Preparation and Characterization of Vinyl Esters

**Figure 1:**
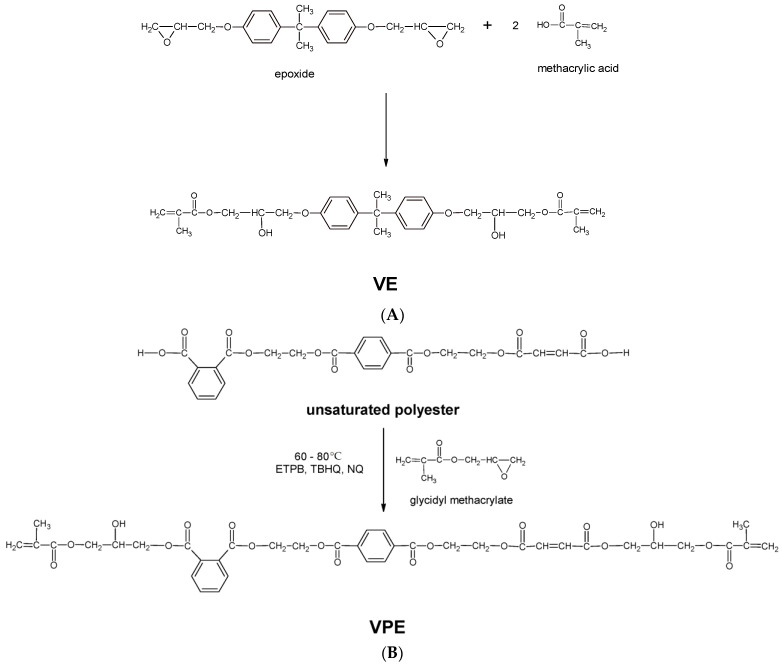
Schematic synthesis of vinyl ester resins based on: bisphenol A (**A**) and unsaturated polyester (**B**).

The schematic synthesis of vinyl ester resins is presented in Figure 1. Vinyl ester resins contain ester groups in the structure of the ester chains and vinyl groups at the ends of the chains. Their exceptional resistance features, which distinguish them from ordinary polyesters, result from the use of bisphenol-A derivatives for their synthesis (Figure 1A).

Bisphenol A (BPA) is a precursor in the production of VERs [31,32]. There is growing evidence that BPA can have adverse effects on human health [10,33,34]. Recent studies suggest that bisphenol A exposure in adults may be associated with reduced ovarian response, miscarriage or premature birth, reduced sexual function in men, altered sex hormones and thyroid levels, and cardiovascular disease [34,35,36,37].

In order to eliminate toxic bisphenol A and, at the same time, obtain an analog of vinyl ester resin, a decision was made to synthesize it based on available polyesters. The process involved the addition reaction of glycidyl methacrylate to the carboxyl groups of polyester with a large acid number. In our research, the polyester was obtained in the polycondensation reaction of phthalic and maleic anhydrides with bis(2-hydroxyethyl)terephthalate (BHET), a hydroxyl-containing compound supplied by Lerg S.A., a company professionally engaged in the production of unsaturated polyester resins. (Figure 1B).

**Figure 2:**
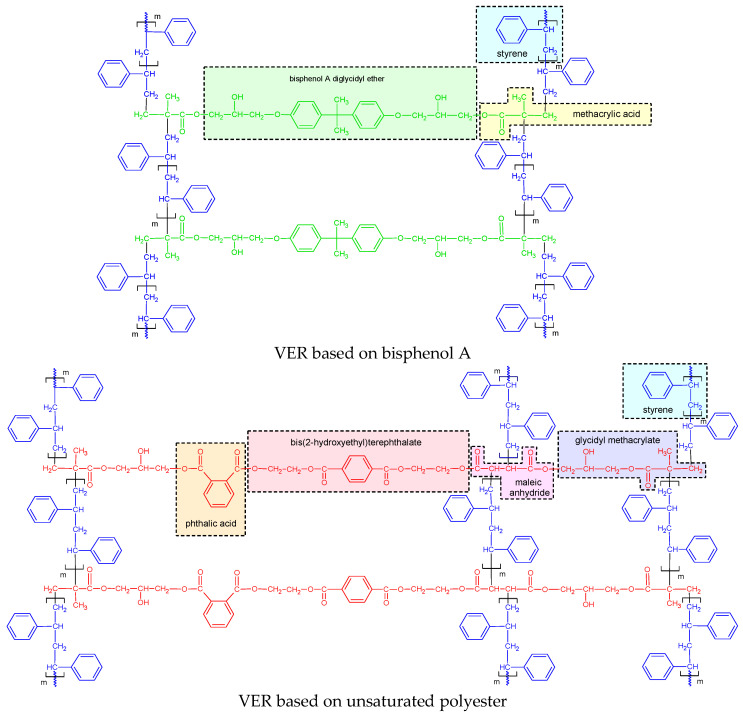
Chemical structures of cured vinyl ester resins.

The results of the chemical structure studies of the resins used for composite preparation are given in Figure 2. Spectrometric identification of molecular organic structures was based on Silverstein’s textbook [38].

**Figure 3:**
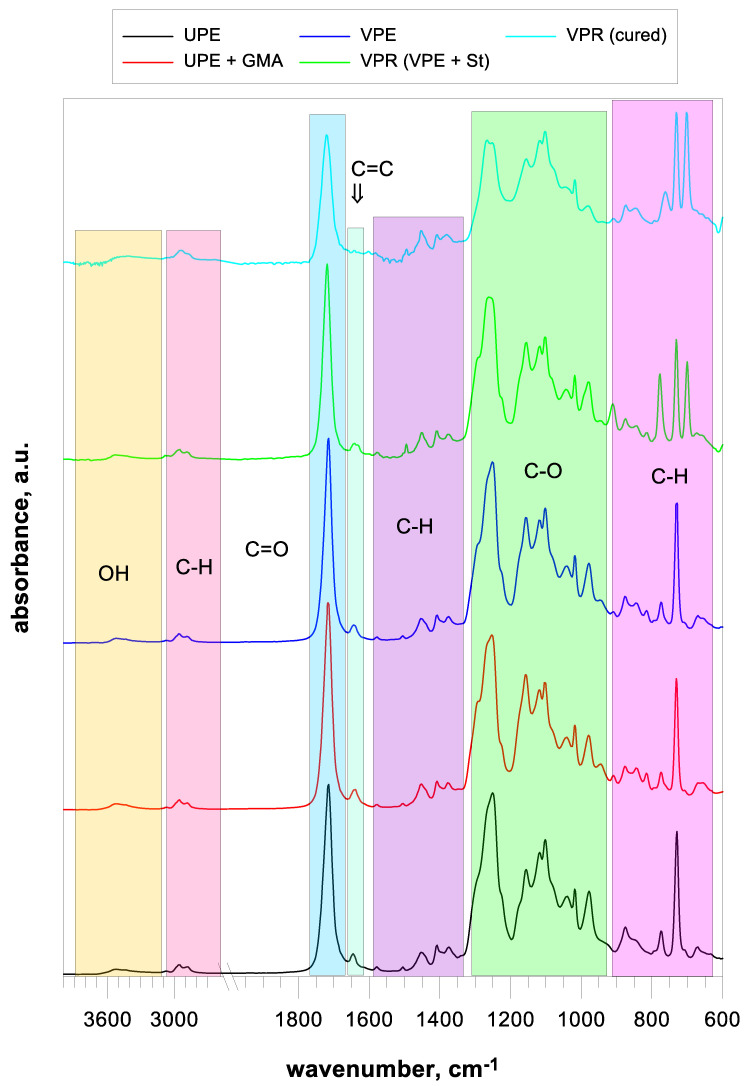
FT-IR spectra of the synthesis stages.

The FT-IR spectrum of the starting unsaturated polyester (UPE) is presented in Figure 3. A very broad peak of 3400–2400 cm^−1^ corresponds to the OH vibrations of the carboxyl end groups of the polyesters. Characteristic absorption bands corresponding to the C-H stretching of the aromatic ring are located above 3000 cm^−1^, while symmetric and anti-symmetric aliphatic stretching occurs at 2956 and 2882 cm^−1^.

The main absorption bands corresponding to the PET structure were confirmed by the vibrations of the bands at 1715 cm^−1^ (C=O stretching), 1250 cm^−1^ (asymmetric stretching of the C-O-C ester), 1156 and 1118 cm^−1^ (para bis substituted aromatic ring), and 1041 cm^−1^ (symmetric stretching of the C-O-C ester). Multiple C-O and C-O-C stretching appeared in the spectrum at 1102, 1018, and 910 cm^−1^. The peak at 730 cm^−1^ corresponds to the CH out-of-plane scissoring vibration of the phenyl ring in bis(2-hydroxyethyl) terephthalate.

The absorption bands of fumarate, originating from the maleic fragment, are located at 1645 cm^−1^ (C=C stretching) and 978 cm^−1^ (trans C=C wagging).

The characteristic bands for C-C and CH vibrations in the rings are presented at 1578 cm^−1^ (phenyl ring C=C), 1505, and 1408 cm^−1^ (aromatic C-H and C-C bonds stretching the phenyl ring). In contrast, the bands at 1452 and 1375 cm^−1^ correspond to symmetric and asymmetric C-H bending vibrations. Three characteristic absorption peaks at 774, 729, and 672 cm^−1^ are related to the C-H out-of-plane deformation vibration.

Infrared spectroscopy was the basic tool to confirm the course of modification of unsaturated polyester. In the FT-IR spectrum of UPE + GMA, additional absorption bands appear when glycidyl methacrylate is added to unsaturated polyester.

The band at 3077 cm^−1^ is associated with the asymmetric =CH stretching of the vinyl group, while the peaks at 910 and 845 cm^−1^ correspond to the vibration bands of the epoxy ring from GMA.

As the addition reaction of glycidyl methacrylate to the terminal carboxyl groups of polyester proceeds, the intensity of the hetero-ring bands decreases gradually. Successful modification of the unsaturated polyester was confirmed by almost complete disappearance of characteristic epoxy bands as well as an increase in the intensity of the C-O stretching vibration peak and the appearance of a peak at 947 cm^−1^ characteristic of the methacrylate group (Figure 3, VPE). Moreover, the wavenumber value corresponding to OH and the band shape were shifted due to the transformation of the polyester end groups. The band from the unsaturated fumarate bond still appears in the spectrum in an unchanged form. This confirms that an additional reaction, not polymerization, has occurred.

After curing with a crosslinking monomer (styrene), characteristic bands originating from the polystyrene fragments can be observed in the FT-IR spectrum (Figure 3, VPR). The bands corresponding to the =C-H stretching caused by the aromatic ring, the peaks at 2924 and 2846 cm^−1^ assigned to the symmetric and asymmetric –CH_2_– stretching vibrations, and the bands at 1602, 1490, and 1447 cm^−1^ corresponding to the stretching vibrations of the C=C bond on the benzene ring are observed. The band at 902 cm^−1^ is assigned to the out-of-plane bending vibrations of the benzene C-H ring.

The decrease in the intensity of the unsaturated bond bands from fumarate and the vinyl terminal bonds from methacrylate clearly confirms the crosslinking of the resin.

The ^1^H NMR proton spectrum of the starting unsaturated polyester (UPE), used for preparation of the VPE resin reveals the characteristic peaks corresponding to the polyester backbone: aromatic protons of the terephthalate unit appear in the region of 7.8–8.2 ppm, methylene protons adjacent to ester linkages (–CH_2_–O–C=O) in the range of 4.1–4.4 ppm, while methylene protons near hydroxyl end groups (–CH_2_–OH) show resonances around 3.6–3.8 ppm. The signal for the vinyl protons from the fumarate unit is observed between 6.2 and 6.5 ppm (trans –CH=CH–).

Following the reaction of UPE with GMA, several distinct changes appear in the proton spectrum. The disappearance of epoxy protons in the 2.6–3.2 ppm region confirms that the oxirane ring in GMA has undergone nucleophilic addition, most likely with terminal –COOH or –OH groups in UPE. Additionally, new signals appear in the range of ~3.2–4.5 ppm, typically as multiplets, which are attributed to methylene and methine protons adjacent to the newly formed ester or ether linkages from the addition reaction. Meanwhile, the signals corresponding to the maleate/fumarate moiety, observed ~6.3 ppm, remain unchanged, indicating that the unsaturation of the polyester is preserved.

The ^13^C NMR spectrum of the base unsaturated polyester (UPE) shows characteristic signals corresponding to its structural units: 165–172 ppm of ester carbonyl carbons (–COO–) from terephthalate and maleate/fumarate, 128–135 ppm of aromatic ring C=C (terephthalate), 132–137 ppm of vinyl C=C from maleate/fumarate, 60–66 ppm of aliphatic carbons adjacent to oxygen (–CH_2_–O–), and 30–40 ppm of internal aliphatic methylene carbons.

The carbon NMR spectrum of the VPE shows several critical changes indicating a successful addition reaction. The disappearance of the epoxy carbons in the 44–50 ppm range signifies the opening of the epoxy ring, providing strong evidence of a chemical reaction between the epoxy group in GMA and the terminal groups in UPE. New signals appearing at 70–75 ppm are characteristic of carbon atoms in secondary alcohol or ether carbon environments, suggesting the formation of covalent bonds between GMA and UPE as a result of the epoxy ring opening. A slight shift in the methacrylate carbonyl signal near 166 ppm indicates that the ester functionality of GMA remains intact, which is consistent with an addition reaction. Furthermore, the signals corresponding to the aromatic and vinyl C=C carbons, specifically from fumarate (~132 ppm) and terephthalate (~128–135 ppm) moieties, remain unaffected, confirming that the polymer backbone is preserved and that the addition of GMA occurs primarily at the terminal sites of the polyester chain.

### 4.2. Properties of Wood Flour Composites

The flexural properties of wood flour (WF) containing composites with different amounts of wood flour are given in Table 1. The same trend was observed for both polymer matrices. The flexural strength and strain at break of WF-reinforced resins decreased with the increasing WF addition, while the flexural modulus and hardness increased.

The pure VER and VPE resins showed the flexural modulus ($E_{f}$) of 3.84 and 3.49 GPa, respectively. The addition of WF increased the modulus of the composite compared to unmodified VER and VPE, which indicates a positive effect on the composite stiffness.

The addition of WF affected the flexural strength ($\sigma_{f}$) of the composites significantly. The flexural strength values of WF-resin followed the opposite trend to $E_{f}$, where the $\sigma_{f}$ of the pure VER decreased from 140 to 67 MPa after loading with 5% WF. The flexural strength of the pure VPE changed from 139 to 69 MPa after loading with 5% WF. The filler plays a key role in the overall flexural strength of the resulting composites.

Strain at break is another parameter describing the flexural properties of materials. The addition of wood flour causes the same value-reducing effect. For pure VER, it changes from 4.08% to 1.79% for VER + 5WF, and for pure VPE and VPE + 5WF from 5.28 to 1.94%.

**Figure 4:**
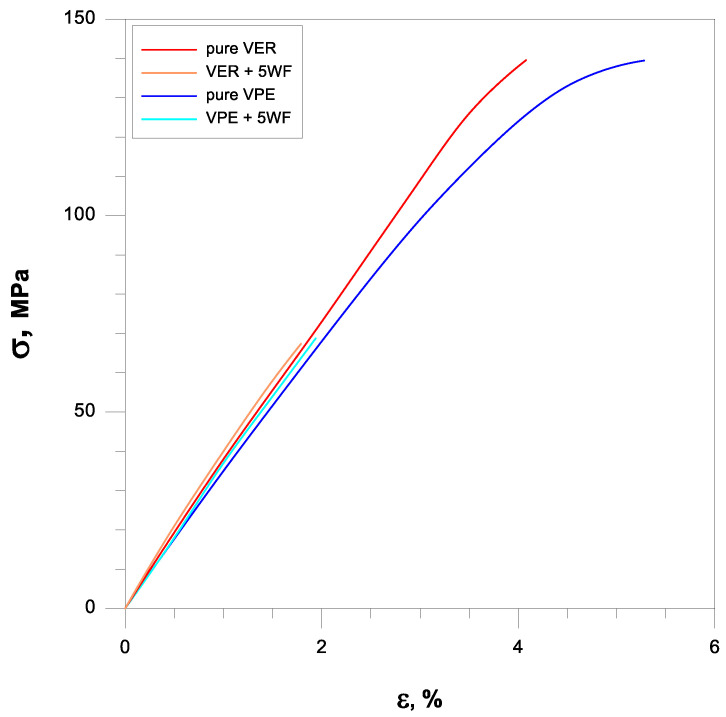
Stress–strain curves of the vinyl ester materials.

Figure 4 shows representative flexural stress–strain curves of the vinyl ester resins and WF composites. Their flexural strength and modulus are summarized in Table 1. Wood flour acts as a rigid, non-elastic filler, causing stress concentration. Hence, materials with WF become more brittle and fail quickly.

The addition of wood flour to the resins affected the hardness of the composites. Pure VER was characterized by the hardness of 80.3 ShD, and with the increase in the amount of filler, this value increased steadily to 81.7 ShD for the material reinforced with 5% WF. The situation for the second resin is analogous and has the same trend, where the pure VPE resin has 78.9 ShD, and the most filled composite has 80.5 ShD.

The results of the Charpy impact tests (Table 1) clearly show that the addition of wood flour (WF) to both vinyl ester matrices (VER and VPE) leads to a significant decrease in impact strength compared to the pure resins.

Dynamic mechanical analysis (DMA) was performed to investigate the effect of wood flour loading on the viscoelastic and thermomechanical behavior of vinyl ester resin (VER and VPE) composites. The storage modulus ($E^{\prime }$), loss modulus ($E^{\prime \prime }$), damping (loss) factor (tan δ), glass transition temperature ($T_{g}$), and crosslinking density ($\nu_{e}$) were evaluated over a temperature range from −150 to 200 °C.

At 20 °C, the $E^{\prime }$ values for all WF-filled composites were higher than those of their respective pure matrices, indicating enhanced stiffness due to the incorporation of rigid wood flour particles. For VER-based systems, $E^{\prime }$ increased from 4.22 GPa (pure VER) to 4.34 GPa (VER + 5WF), and a similar trend was observed for VPE-based systems (from 3.84 GPa to 3.94 GPa). The effect of WF was more pronounced at elevated temperatures. At 180 °C, the storage modulus of VER + 5WF reached 14.27 MPa, compared to 15.44 MPa for the pure VER, suggesting improved dimensional stability in the rubbery plateau region.

The loss modulus ($E^{\prime \prime }$), which reflects the material’s ability to dissipate mechanical energy, decreased with increasing WF content (Table 2). The $E^{\prime \prime }$ value decreased significantly with WF addition in both resin systems: from 220 MPa (pure VER) to 104 MPa (VER + 5WF), and from 202 MPa (pure VPE) to 95 MPa (VPE + 5WF). This reduction corresponds well with the observed decline of Charpy impact strength (Table 1), indicating reduced molecular mobility.

The tan δ provided further insight into the damping characteristics and molecular mobility of the composites. The height of the tan δ peak, which measures damping, decreased with WF addition, reflecting reduced polymer segmental motion due to physical confinement by the filler. For instance, the tan δ peak of VER decreased from 0.85 to 0.55 (pure VER to VER + 5WF), and that of VPE changed from 0.78 to 0.52 (pure VPE to VPE + 5WF), consistent with more restricted chain movement. In parallel, the full width at half maximum (FWHM) of the tan δ peak also narrowed, indicating a sharper and more brittle transition, in agreement with the decreasing strain at break observed in the mechanical test.

The $T_{g}$ values, determined from the tan δ peak, showed a slight decrease with increasing WF content (Table 2), shifting from 120 °C (pure VER) to 109 °C (VER + 5WF), and from 125 °C (pure VPE) to 112 °C (VPE + 5WF). This trend suggests that the filler restricts chain mobility.

The crosslinking density ($\nu_{e}$) of the composites was estimated from the storage modulus in the rubbery plateau region using rubber elasticity theory, providing valuable insights into the polymer network structure and its interaction with fillers. Since VERs contain fewer C double bonds than unsaturated polyesters, their crosslinking density is typically lower than that of UPRs [7]. This phenomenon can also be explained by the fact that the reactive double bonds are located at the ends of relatively long chains [31].

As shown in Table 2, the pure VER resin exhibited a crosslinking density of 1365 mol m^−3^, which decreased to 1264 mol m^−3^ upon addition of 5 wt% wood flour (WF). A similar trend was observed for the VPE composites, where the crosslinking density dropped from 1399 mol m^−3^ for the pure VPE to 1295 mol m^−3^ with the incorporation of 5 wt% WF. The VPE materials were characterized by higher crosslink density values compared to those based on VER. This is due to the fact that the novel vinyl ester resin obtained from unsaturated polyester contains additional C=C bonds derived from maleic anhydride, in addition to the typical vinyl ones at the chain ends.

It is noteworthy that the crosslinking density values of vinyl ester resins (VER and VPE) are lower compared to those of the unsaturated polyester resin reported in the previous study (1431 mol m^−3^), as expected [5,39].

Moreover, the addition of wood flour reduces the volume fraction of the polymer matrix, resulting in an apparent dilution effect that contributes to the observed decrease in crosslink density. This effect is consistent across both VER and VPE matrices, indicating a general influence of wood flour on network formation regardless of the resin type.

The DMA results align closely with the trends observed in the mechanical test. The increase in storage modulus parallels the increase in flexural modulus with WF content (Table 1), while the reduction in loss modulus and tan δ is consistent with the decrease in Charpy impact strength and strain at break.

**Figure 5:**
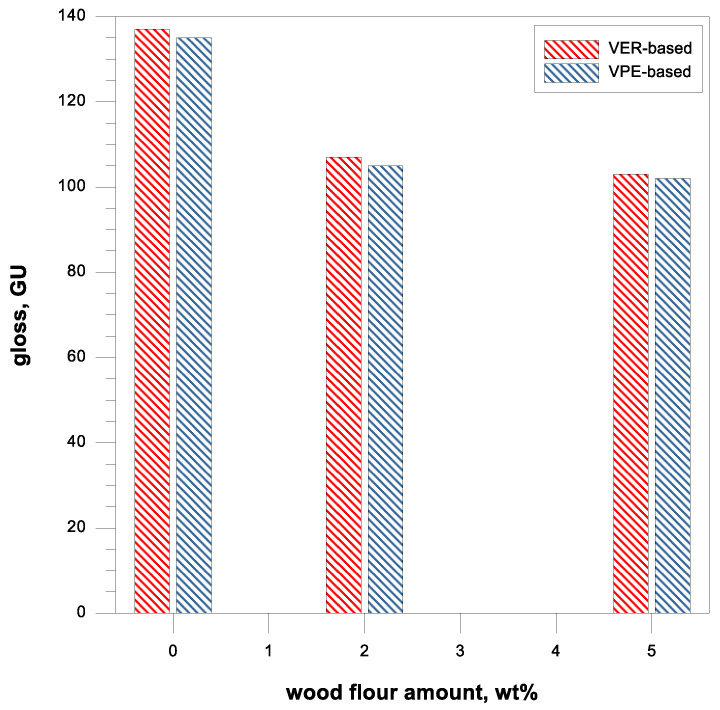
Gloss of the studied samples.

The results of gloss measurements of crosslinked resin samples and their composites with wood flour are presented in Figure 5. The Zehntner ZGM 1110 glossmeter used to test surfaces allowed the determination of this parameter, when measuring simultaneously at the geometric configurations of 20°, 60°, and 85° of light incidence, values corresponding to a high-gloss or matt surface were obtained. As the reference standard, highly polished black glass with the gloss of 86.8 (20°), 93.4 (60°), and 99.7 GU (85°) was used.

From the data obtained, it can be concluded that with the addition of wood filler, the gloss decreases. This phenomenon is very well known and observed for polymers [25]. In the previous paper, where the unsaturated polyester resin (UPR) was used as the polymer matrix, a material with a gloss of 112.4 GU was obtained [25]. For its composites with 2 and 5 wt% of wood flour, the values of this parameter were 101.8 and 95.3 GU, respectively.

Vinyl ester resins are characterized by much better gloss, and the values are quite similar to each other in the range of 135–137 GU. It is worth noting that the VPE obtained from unsaturated polyester, the same as in UPR, was characterized by gloss comparable to the commercial vinyl ester resin based on bisphenol A. As mentioned earlier, resin-wood materials were characterized by smaller glosses in relation to pure resins. The decrease in the gloss value is caused by obtaining an uneven surface due to the presence of filler particles. VER materials with 2 and 5 wt% of wood flour had the gloss 107 and 103 GU, respectively, while the VPE samples, 105 and 102 GU. Nevertheless, vinyl ester resin composites based on bisphenol A, as well as unsaturated polyester, showed gloss at geometry 60° significantly larger than 70 GU, confirming the belief that materials with high surface gloss were obtained.

In order to investigate the potential applications of the tested resins and their composites with wood flour for biomedical purposes, it was necessary to examine how the presence of a biofiller would affect the wettability of their surfaces. For this purpose, surface contact angle tests were carried out using distilled water (Table 3). Contact angle analysis is a convenient tool used for the determination of the quality of a solid surface. The results show that for pure VER the contact angle measured with water is 73.3°, while its values decrease with increasing wood flour concentration. For the sample containing 5% wood flour, it decreased to 68.2°.

A similar trend can be observed for the VPE resin and its composites, except that the contact angle of water for pure resin is much larger than for VER, being 89.9°. Adding wood flour to the resin caused a decrease in the contact angles of the surfaces of the obtained composites. For the composite with 2 wt% of WF, it was 80.2°, and for that with 5 wt% of WF, it was 76.8°.

Larger contact angles obtained for the VPE resin and its composites indicate that their surfaces have small wettability—that is, the water droplet will not spread out much on the surface. The obtained results indicate that the VPE resin is much more hydrophobic than the VER resin. Similarly, the composites obtained from it with WF also have larger hydrophobic surfaces.

As expected from our studies, vinyl ester resins, due to their lower polarity and higher hydrophobicity, have a larger contact angle with water than unsaturated polyester resins (57.0°), and therefore are characterized by lower surface wettability [39].

### 4.3. Biomedical Application

From the perspective of manufacturing biomedical products, VPE resin appears to be a more suitable matrix than VER resin, as it presents a lower probability of colonization by microorganisms that require moisture for growth.

When evaluating materials intended for the medical, pharmaceutical, cosmetology, or food industries, it is crucial to assess their resistance to bacterial biofilm adhesion. For this purpose, a biofilm formation test was performed on the surface of modified and unmodified materials. The results obtained were compared with the biofilm formed on the bottom of a well in a 24-well polystyrene plate, which served as the control.

The bacterial strains used in this test—*S. aureus* ATCC 25923 or *E. faecalis* PCM 896, or *E. coli* ATCC 25992—are commonly associated with infections in medical and food-related industries. Planktonic bacterial cells were cultured under appropriate conditions for 48 h (double incubation) to allow the development of a mono-species biofilm.

In the qualitative test, after culturing the biofilm and rinsing away planktonic cells from the materials, the bacterial cells comprising the biofilm were stained. Using a live/dead staining method, it was possible to determine the structure of the resulting biofilm in terms of cell viability. Specifically, living cells were stained green, while the dead cells were stained red.

**Figure 6:**
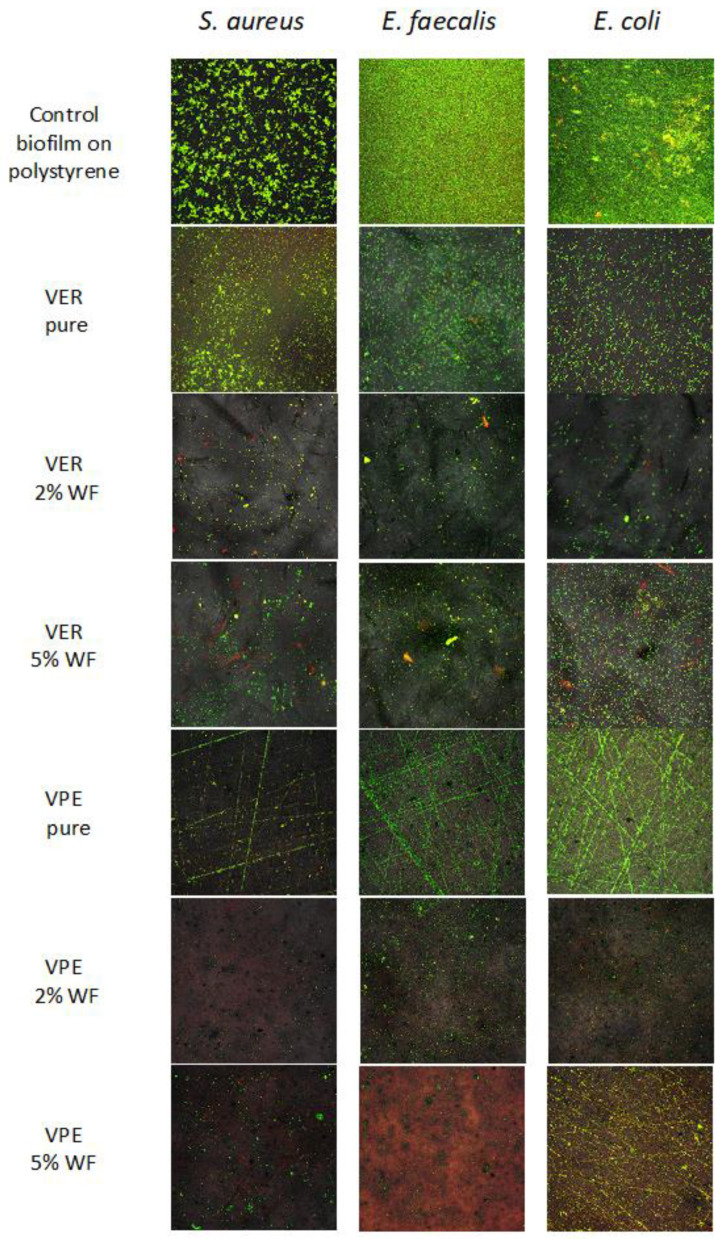
Visualization of the CLSM images of biofilm formed by *S. aureus* ATCC 25923 or *E. faecalis* PCM 896, or *E. coli* ATCC 25992 on the surface of the tested materials. (Magnification 200×, green fluorescence—viable cells, red and yellowish fluorescence—dead bacterial cells).

The images obtained show that each of the tested strains, both Gram-positive and Gram-negative, formed extensive biofilm on the control 24-well plates, consisting exclusively of live (green) cells. The biofilm formed as a distinct monolayer with a spatially extensive architecture (Figure 6).

**Figure 7:**
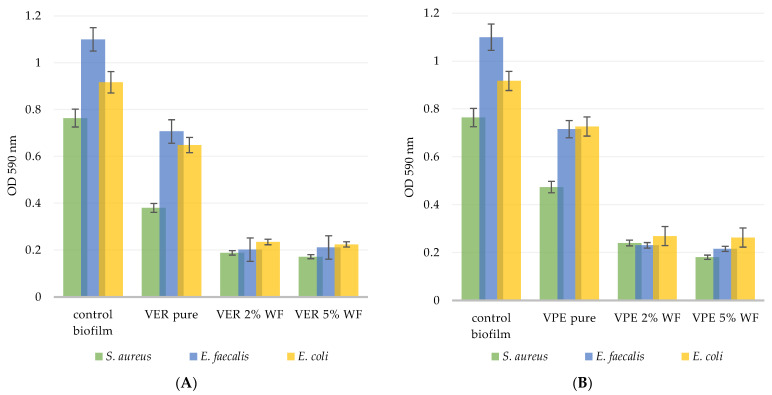
Quantification of the bacterial cells adhered to the tested wood materials: (**A**) VER pure, VER 2%, VER 5% and (**B**) VPE pure, VPE 2%, VPE 5% compared to the control polystyrene surface (polystyrene of a 24-well plate).

Furthermore, the images in Figure 7 show that on pure VER and VPE resins, all Gram-positive and Gram-negative strains exhibited much weaker adhesion and did not form a monolayer, in contrast to the biofilm growth on polystyrene control. On the pure materials, biofilm appeared only locally and was limited to the areas where the sample was scratched or created wrinkles, as visible in the confocal microscope images. The VPE samples exhibited more such wrinkles than the VER one.

Importantly, both VER and VPE materials containing 2 wt% and 5 wt% of wood flour were essentially free of biofilm, with only dead (red) bacterial cells or occasional planktonic living cells observed. This effect was particularly evident for *E. faecalis* on the VPE composite containing 5 wt% wood flour. Regardless of the amount of wood filler (2 wt% or 5 wt%), the biofilm images were comparable, showing no biofilm formation. These results indicate that the composites are more resistant to bacterial colonization compared to the pure resins, a finding that applies to both VPE and VER materials.

Another experiment was performed to quantify biofilm formation depending on the type of composite used. Figure 6 confirms the results of the previous experiment, showing that all tested materials—both VER and VPE—exhibited anti-biofilm activity. Modification of the materials with wood flour, regardless of the filler content (2 wt% or 5 wt%), appeared effective, as the VER and VPE composites demonstrated greater resistance to biofilm formation than the unmodified pure VER and pure VPE, which served as the initial controls.

Precisely, both VER– and VPE–composites were more resistant to bacterial colonization by the Gram-positive strains than by the Gram-negative *E. coli*.

The VER material exhibited a more favorable structure and composition than VPE, inhibiting the growth of *S. aureus* by 75.4% and 77.5% for the 2% and 5% WF composites, respectively; *E. faecalis* by 81.8% and 80.9%; and *E. coli* by 72.3% and 74.6%, respectively.

For VPE, the percentage of biofilm inhibition for 2% and 5% WF modifications was as follows: *S. aureus*—68.6% and 76.5%; *E. faecalis*—79.1% and 80.4%; *E. coli*—68.9% and 71.4% (Figure 6).

Although there are differing opinions among researchers regarding the ability and strength of biofilm formation depending on the surface, all authors agree that biofilm adhesion is influenced by several factors: surface roughness (micropores, unevenness, and general roughness increase the surface area available for bacterial attachment), hydrophobicity (hydrophobic surfaces promote adhesion), electrostatic interactions (positively charged surfaces attract negatively charged bacteria), and the presence of potential nutrients in the material composition.

Park et al. focused on the influence of surface roughness on bacterial biofilm formation. The authors conducted experiments to investigate how different degrees of roughness affect bacterial adhesion and biofilm development [40]. Similarly, Sorongon et al. focused on the role of surface hydrophobicity in biofilm formation on the surfaces in contact with food. The authors carried out research to investigate how different surface hydrophobic properties influence bacterial adhesion and biofilm growth [41].

The biofilm formation results indicate that the VPE resin exhibits better antibiofilm properties. This is due to the different chemical structure of the matrix, which was previously confirmed by surface contact angle studies, indicating its more hydrophobic nature.

*Staphylococcus aureus* is a Gram-positive bacterium that can cause infections of the skin, wounds, soft tissue, and the respiratory system [42]. It is capable of forming a durable biofilm on medical surfaces such as prostheses, catheters, and implants.

*Enterococcus faecalis* is naturally present in the human digestive tract. However, it can cause urinary tract and wound infections as well as other nosocomial infections [43]. It is also capable of forming a biofilm on the surfaces of devices used in medical, beauty, and cosmetology settings.

On the other hand, *Escherichia coli* is a Gram-negative bacterium commonly found in human intestines. Certain strains can be pathogenic, causing urinary tract and other infections [44]. *E. coli* can spread in public places if hygiene is not maintained. Bacterium is known to form biofilms on medical and dental surfaces, such as catheters and dentures.

Cytotoxicity tests revealed that the tested materials based on different vinyl ester resins were non-toxic to skin fibroblasts. The WST-8 test showed that both the native and modified variants (pure or WF-enriched) had no adverse effects on normal human fibroblasts after both 24 h and 48 h exposure to 24 h extracts. However, the cell viability after exposure to 48 h extracts of VER 5%WF was reduced to approximately 76% compared to the polystyrene control, likely due to the higher amount of wood flour in 48 h extracts. It is worth noting that according to the ISO 10993-5, all tested materials are considered non-toxic, as cell viability did not fall below 70% [29].

**Figure 8:**
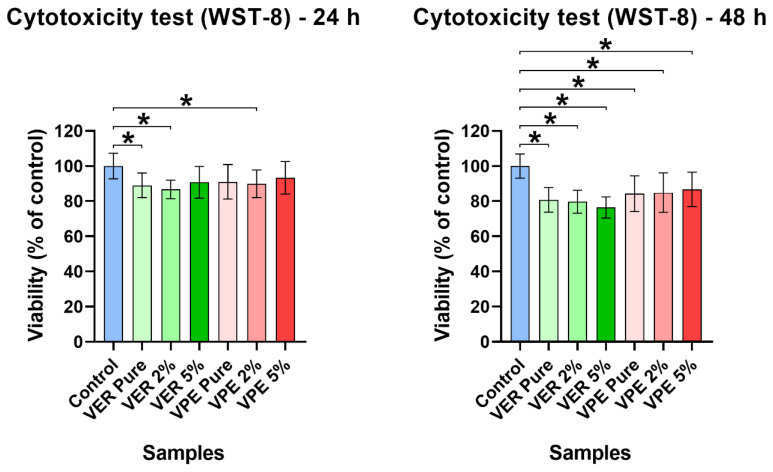
BJ cell viability after 24 and 48 h of incubation at 37 °C using the WST-8 test.

Although both polymer matrices exhibited similar cell viability overall, as shown in Figure 8, the synthesized vinyl ester resin based on unsaturated polyester (VPE) performed slightly better than the commercially available vinyl ester resin derived from the epoxy derivative of bisphenol A (VER).

**Figure 9:**
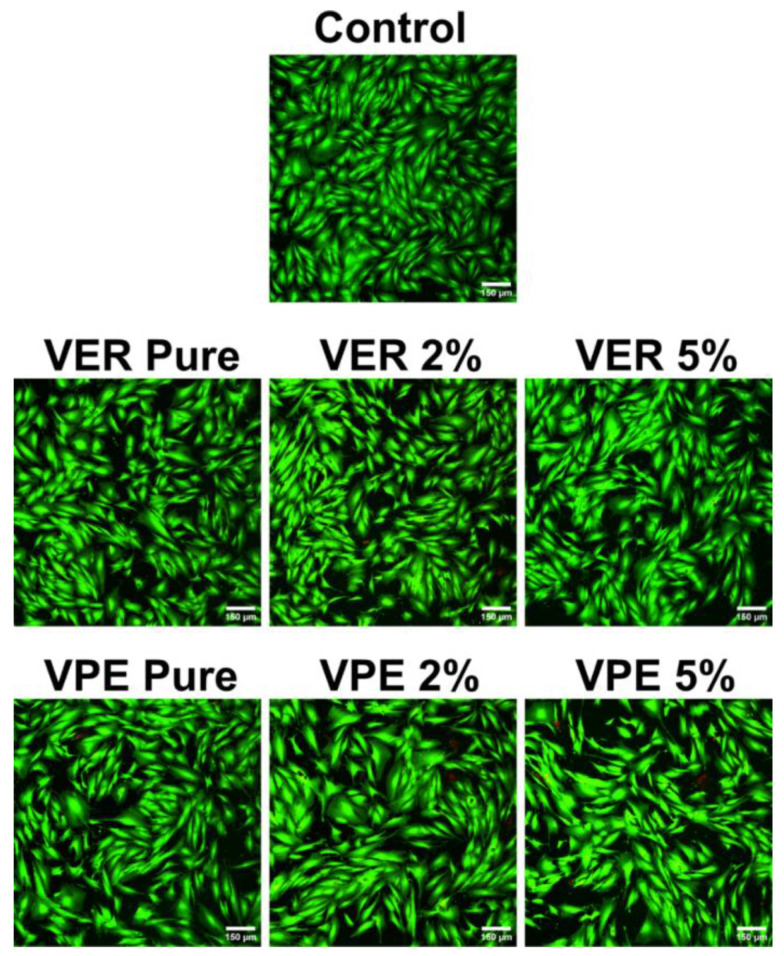
Live/Dead assay of fibroblast in contact with the PS control, pure VER, pure VPE, and their composites with different amounts of wood flour.

Cytotoxicity of the resin materials was also assessed using the indirect contact method with Live/Dead fluorescent staining of BJ fibroblasts grown in the presence of 24 h extracts of the tested materials. CLSM images revealed a healthy monolayer of viable cells with typical spindle-shaped morphology after exposure to all tested extracts, as shown in Figure 9, confirming the non-toxic nature of the materials. The viability and morphology of cells exposed to the extracts were comparable to those of the control cells grown in fresh culture medium, which is particularly noteworthy.

## 5. Conclusions

The paper presents a new method of synthesis of vinyl ester resin, which does not contain bisphenol A derivative fragments in the structure. The obtained resin served as a matrix in preparation of composites with wood flour from spruce (*Picea*) and fir (*Abies*). Composites with commercially available resin containing bisphenol A fragments in the structure were obtained in an analogous manner. Their properties were studied and compared. Due to possible biomedical applications, emphasis was put on their bactericidal properties and cytotoxicity. Differences in the chemical structure of the matrices make the VPE resin more hydrophobic and therefore have better anti-biofilm properties. The results obtained for skin fibroblasts indicate the non-toxicity of both polymer matrices, confirming that the synthesized BPA-free vinyl ester resin shows improved cytocompatibility. Studies of the resistance of these composites to bacterial biofilm adhesion to Gram-positive and Gram-negative bacterial strains showed significantly weaker adhesion, and no monolayer was formed, compared to the control biofilm growth on polystyrene. It is important that both VER and VPE materials containing 2 and 5% wood flour were free of biofilm, which means that WF composites have greater prospects for biomedical applications than the original resins.

Overall, the addition of wood flour to vinyl ester resin composites enhances stiffness and crosslinking density while reducing damping and impact toughness. The shift in glass transition temperature and increased modulus in the rubbery region indicate more thermally stable network structures, albeit with reduced energy absorption capabilities. These findings provide important design guidelines for developing bio-based thermoset composites with tailored mechanical and thermal properties.

## Figures and Tables

**Table 1:** Mechanical properties, hardness, and Charpy impact strength of resin-based composites with wood flour.

*Sample*	$E_{f}$ ^1^, GPa	$\sigma_{f}$ ^2^, MPa	$\varepsilon_{f}$ ^3^, %	HD ^4^, ShD	$a_{cU}$ ^5^, kJ m^−2^
pure VER	3.84 ± 0.06	139.59 ± 6.94	4.08 ± 0.08	80.3 ± 0.3	17.90 ± 2.44
VER + 2WF	3.89 ± 0.02	88.10 ± 4.90	2.32 ± 0.14	80.8 ± 0.4	4.92 ± 0.67
VER + 5WF	4.07 ± 0.05	67.40 ± 1.66	1.79 ± 0.10	81.7 ± 0.3	3.40 ± 0.23
pure VPE	3.49 ± 0.03	139.47 ± 6.72	5.28 ± 0.13	78.9 ± 0.2	12.50 ± 1.92
VPE + 2WF	3.52 ± 0.01	89.86 ± 5.08	2.61 ± 0.17	79.6 ± 0.4	7.96 ± 0.88
VPE + 5WF	3.58 ± 0.01	68.79 ± 2.05	1.94 ± 0.06	80.5 ± 0.3	4.04 ± 0.21

^1^ Flexural modulus; ^2^ Flexural strength; ^3^ Strain at break; ^4^ Shore hardness D; ^5^ Charpy impact strength.

**Table 2:** Thermomechanical parameters of resin-based composites with wood flour obtained by DMA.

*Sample*	$E^{\prime }$(*20* °C) ^1^, GPa	$E^{\prime }$(*180* °C) ^1^, MPa	$E^{\prime \prime }\;$^2^, MPa	tan δ ^3^	FWHM ^4^, °C	$T_{g}\;$^5^, °C	$v_{e}\;$^6^, mol m^−3^
pure VER	4.22	15.42	220.03	0.85	25	120	1364.9
VER + 2WF	4.26	14.81	162.87	0.68	22	114	1311.3
VER + 5WF	4.34	14.27	104.12	0.55	19	109	1263.5
pure VPE	3.84	15.79	202.14	0.78	27	125	1398.6
VPE + 2WF	3.87	15.24	144.67	0.65	24	117	1349.7
VPE + 5WF	3.94	14.63	95.87	0.52	22	112	1295.3

^1^ Storage modulus, glassy state and rubbery plateau; ^2^ Loss modulus; ^3^ Loss factor; ^4^ Full width at half maximum; ^5^ Glass transition temperature from tan δ curve; ^6^ Crosslinking density.

**Table 3:** Droplet and value of contact angle measurements with distilled water of wood-resin composites.

*Sample*	*VER*	*VPE*
pure	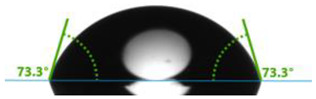	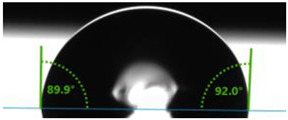
2 wt% WF	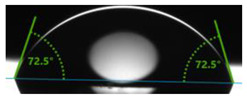	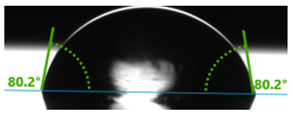
5 wt% WF	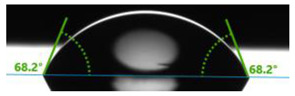	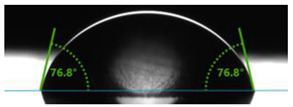

## Data Availability

The dataset is available on request from the authors.
